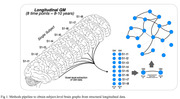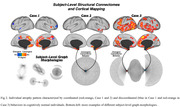# Subject‐level Detection of Focal Neurodegeneration Using Spatiotemporal Connectomics: Towards Atrophy Characterization in Preclinical Alzheimer’s Disease

**DOI:** 10.1002/alz.089064

**Published:** 2025-01-09

**Authors:** Cristina Sánchez, Ibai Diez, Elisenda Bueichekú, Chan‐Mi Kim, Michel J. Grothe, Pascual Sanchez‐Juan, Jorge Sepulcre

**Affiliations:** ^1^ Reina Sofia Alzheimer Center, CIEN Foundation, ISCIII, Madrid, Madrid Spain; ^2^ Gordon Center for Medical Imaging, Massachusetts General Hospital, Boston, MA USA; ^3^ Reina Sofia Alzheimer Centre, CIEN Foundation, ISCIII, Madrid Spain

## Abstract

**Background:**

Brain atrophy is a normal part of healthy aging, but it is aggravated by several neurodegenerative diseases. Previous studies have described a large heterogeneity in individual neurodegeneration patterns, but the underlying brain mechanisms are currently not fully understood. From a graph theory‐based framework, the estimation of subject‐specific focal or multifocal brain atrophy in healthy aging and in the preclinical stage of different neurodegenerative diseases, such as Alzheimer's disease (AD), will help to better understand individual atrophy networks and likely improve prediction of phenotypic heterogeneity in disease trajectories. This study aimed to develop a novel spatiotemporal connectomic method based on graph theory applied to serial MRI measurements to identify neurodegeneration focality (i.e., unifocal or multifocal atrophy patterns) in healthy aging at the single‐subject level.

**Method:**

The study included a unique sample of 79 older cognitively normal participants from the the Vallecas project who underwent longitudinal T1 MRI scanning with 8 follow‐up timepoints over 9,46±1,97 years. Voxel‐based morphometry was used to define the brain atrophy topology of each subject, and uni‐ or multifocal atrophy patterns were identified using a graph theory approach based on structural similarity between each voxel and the rest of the brain across serial gray matter measurements (Figure 1).

**Result:**

We identified individualized atrophy phenotypes characterized by different graph morphologies (Figure 2), which could be classified into three main groups based on their connectivity behavior. One group of subjects was characterized by atrophic voxels with a coordinated behavior, another with a predominance towards divergent or discoordinated behavior, and a third group that laid between these two extremes.

**Conclusion:**

We present a novel analytical tool for characterizing individualized atrophy phenotypes in healthy subjects based on graph theory and structural similarity analyses of longitudinal MRI data. This method may help to describe the first structural events in preclinical AD and other neurodegenerative diseases and, therefore, could be crucial for predicting differences in disease phenotype and progression in single subjects.